# Using Large-scale Social Media Analytics to Understand Patient Perspectives About Urinary Tract Infections: Thematic Analysis

**DOI:** 10.2196/26781

**Published:** 2022-01-25

**Authors:** Gabriela Gonzalez, Kristina Vaculik, Carine Khalil, Yuliya Zektser, Corey Arnold, Christopher V Almario, Brennan Spiegel, Jennifer Anger

**Affiliations:** 1 Department of Urology Davis School of Medicine University of California, Davis Sacramento, CA United States; 2 Cedars-Sinai Center for Outcomes Research and Education Los Angeles, CA United States; 3 David Geffen School of Medicine University of California Los Angeles, CA United States; 4 Computational Diagnostics Departments of Radiology and Pathology University of California Los Angeles, CA United States; 5 Department of Urology University of California San Diego La Jolla, CA United States

**Keywords:** female urology, urinary tract infections, health services research, social media, online community, online forum, latent Dirichlet allocation, data mining, digital ethnography

## Abstract

**Background:**

Current qualitative literature about the experiences of women dealing with urinary tract infections (UTIs) is limited to patients recruited from tertiary centers and medical clinics. However, traditional focus groups and interviews may limit what patients share. Using digital ethnography, we analyzed free-range conversations of an online community.

**Objective:**

This study aimed to investigate and characterize the patient perspectives of women dealing with UTIs using digital ethnography.

**Methods:**

A data-mining service was used to identify online posts. A thematic analysis was conducted on a subset of the identified posts. Additionally, a latent Dirichlet allocation (LDA) probabilistic topic modeling method was applied to review the entire data set using a semiautomatic approach. Each identified topic was generated as a discrete distribution over the words in the collection, which can be thought of as a word cloud. We also performed a thematic analysis of the word cloud topic model results.

**Results:**

A total of 83,589 posts by 53,460 users from 859 websites were identified. Our hand-coding inductive analysis yielded the following 7 themes: quality-of-life impact, knowledge acquisition, support of the online community, health care utilization, risk factors and prevention, antibiotic treatment, and alternative therapies. Using the LDA topic model method, 105 themes were identified and consolidated into 9 categories. Of the LDA-derived themes, 25.7% (27/105) were related to online community support, and 22% (23/105) focused on UTI risk factors and prevention strategies.

**Conclusions:**

Our large-scale social media analysis supports the importance and reproducibility of using online data to comprehend women’s UTI experience. This inductive thematic analysis highlights patient behavior, self-empowerment, and online media utilization by women to address their health concerns in a safe, anonymous way.

## Introduction

Symptomatic acute bacterial cystitis, often used interchangeably with the term urinary tract infection (UTI), affects 60% of women once in their lifetime [1,2]. Based on self-reported data, the National Health and Nutrition Examination Survey identified a 12.6% annual incidence of UTI among women aged 18 years or older [3]. The cost of diagnosing and treating UTI has been estimated to be US $1.6 billion, which does not account for the management of recurrent UTI (rUTI) [1]. Up to 44% of women will have recurrent UTIs, defined as 2 or more UTIs within a 6-month period or 3 or more UTIs within 12 months [4].

Current qualitative studies among women with UTIs focus on prescription practice patterns, self-management strategies, and UTIs during pregnancy [5-7]. These studies are usually conducted in the clinical setting. Leveraging social media to understand the UTI experience of patients remains unexplored, despite the prominent use of online sources to supplement medical care among middle-aged to older adults [8,9].

To understand women’s knowledge and experience with UTIs, we used digital ethnography to investigate patient perspectives via online media [9]. This research method adapts conventional ethnographic principles to understand a phenomenon in a population of interest by allowing investigators to study social media posts and conversations that serve as free-range, nonexperimental sources that may be generalized to a broader population of women with UTI [9]. Using online sources is a nonconventional tool to gather patient perspectives to better meet the medical needs of women with UTI seeking advice outside the clinical setting. Physicians should counsel patients based on their direct concerns. The National Institute of Diabetes and Digestive and Kidney Diseases–funded Prevention of Lower Urinary Tract Symptoms (PLUS) Network supported our efforts to complement its goals of understanding personal and environmental factors affecting women’s bladder health [10]. The PLUS Consortium adopted the social ecological model, which considers interactions between social context and biology across the lifespan and views health behaviors as being determined by intrapersonal factors, interpersonal processes and primary groups, institutional factors, community factors, and public policy. We sought to characterize the awareness, patient experience, prevention strategies, and risk factors among women with UTI by conducting an ethnographic analysis of social media posts.

## Methods

### Data Acquisition

This study was found exempt by our institution’s institutional review board. To gather large-scale online posts by women with UTI, we contracted with Treato, a web-based data-mining service company that utilizes extraction templates and a proprietary search algorithm designed to capture patient content. After consultation with the PLUS Consortium, our team chose a combination of keywords related to disease nomenclature, symptoms, treatment options, and exclusion terms to identify posts using their search algorithm (Multimedia Appendix 1). After extracting posts from online forums, we performed digital ethnography using qualitative thematic analysis and a latent Dirichlet allocation (LDA) topic modeling quantitative process that also facilitated qualitative results [11]. Combining both methods allowed us to ensure thematic saturation by analyzing the entire data set of identified posts.

### Qualitative Analysis

After identifying posts that met our search criteria, the entire data set was randomized to ensure that we reviewed posts from various websites before reaching thematic saturation. We performed an inductive, iterative, open-coding, qualitative analysis until we could no longer identify unique themes. Two research team members were assigned to examine the extracted posts several times. The data were then organized into different units or codes, which provides sufficient detail for the reader even without the context. Therefore, the codes were supported by text fragments. This was an iterative process; hence, as unique inductive codes emerged, they were regrouped into more specific categories, and some were combined while others were placed in a superordinate category. Our goal was to avoid redundancy among the categories, so we created broad themes encompassing the categories.

### LDA: A Quantitative and Qualitative Approach

To supplement the manual inductive coding process, we applied a second, more novel technique, LDA, that allowed for the review of the entire data set. LDA is an unsupervised probabilistic topic model process that relies on the contextual co-occurrence of words to identify patterns of words that, when found together, have a semantic meaning [11,12]. For example, the word “bank” can have different meanings when paired with “money” versus “water.” This model generates outputs as topics that can be understood with the concept of a “word cloud,” comprised of words that are ranked higher in a corresponding topic, if these co-occurred frequently in the social media posts. These topics were interpreted for thematic analysis. Each topic has an assigned prevalence value, which represents the quantity of words in the collection assigned to the topic divided by the total number of words. The word cloud topics were sorted based on their respective prevalence (quantitative signature) for review by the research team to identify a thematic interpretation. Consistency of theme allocation for a specific topic was confirmed by reviewing posts that contributed to the development of the word cloud. Table 1 demonstrates examples of the word cloud topics and their assigned themes. Combining both methods allowed for a comprehensive review of the results using a semiautomated approach and a manual inductive coding process to capture a broad understanding of the experience of patients with UTI.

**Table 1:** Example word cloud topics with their respective prevalence and assigned themes.

Topics	Prevalence (%)	Themes
uti, burning, symptoms, urination, urine, urinate, frequent, sensation, urinating, urge, ago, having, past, started, feel, time, discomfort, painful, year, week	15	Online community support (symptom sharing)
years, utis, recurrent, months, year, ago, antibiotics, infections, cause, menopause, sex, antibotics, colonized, time, month, bladder, mg, antibiotic, uti, stopped, tried, chronic	14	Recurrent UTI^a^, risk factors and prevention
uti, taking, antibiotics, antibiotic, prescribed, mg, infection, took, macrobid, bactrim, taken, week, amoxicillin, started, medication, nitrofurantoin, got, pills, allergic, prescription	11.5	Antibiotics
use, clean, shower, utis, soap, uti, using, underwear, make, baths, used, cause, infections, toilet, utis, skin, sure, wipe, change, hot water, douche	10.5	Hygiene, risk factors and prevention
infection, uti, antibiotics, need, kidneys, bladder, cause, utis, infections, untreated, symptoms, treated, pregnancy, worse, sounds, checked, definitely, serious, asap, away	5	Treatment (untreated UTI)
bladder, cystitis, ic, symptoms, urologist, interstitial, years, flare, diagnosed, help, diet, chronic, uti, condition, infection, painful, urethra, thought, inflammation, pelvic, misdiagnosed	4	Diagnosis (overlap with interstitial cystitis)
cranberry, drink, juice, uti, drinking, help, lots, utis, make, helps, utis, sure, pills, fluids, antibiotics, flush, bladder, need, avoid, drinks	3.5	Alternative therapies

^a^UTI: urinary tract infection.

## Results

We identified 83,589 posts written by 53,460 unique users found on 859 websites from January 2016 to December 2018.

### LDA Topic Modeling Themes

We identified a total of 105 themes using LDA, which were grouped into 9 categories to avoid redundancy and provide an overview of the topics represented online (Table 2). Additionally, there was significant overlap with our hand-coding approach, so the data were synthesized into 7 themes with subthemes (Table 2) to represent results from both methods. Our hand-coding approach facilitated more descriptive interpretations.

**Table 2:** Nine categories of themes found using latent Dirichlet allocation.

Categories	Prevalence (N=105), n (%)
Online community support	27 (25.7)
Risk factors and prevention	23 (22.0)
Self-management strategies	14 (13.3)
Antibiotics	11 (10.5)
Alternative therapies	7 (6.7)
Access to care	7 (6.7)
Treatment options	7 (6.7)
Quality of life	6 (5.7)
Diagnosis	3 (2.9)

### Inductive Thematic Analysis

Qualitative hand-coding analysis yielded 7 themes with subthemes related to the knowledge and experience of women with UTI symptoms (Table 3).

**Table 3:** Themes and illustrative examples from 200 online posts.

Theme	Subtheme	Example quotes
Quality of life	Impact on sexual health Fear Pain Self-blame	“UTIs always dictate my life and make me feel very low, I’m always worried about going out for the day and whether there will be toilets around” “feeling helpless, like it’s a nightmare: rUTIs”
Knowledge acquisition	Differential diagnosis UTI^a^ workup Seeking an etiology IC^b^ symptom overlap Untreated UTIs	“I was told I had trace blood in urine…what’s trace?... Dr. didn’t explain.” “Some of what I thought was constant UTIs was actually interstitial cystitis - an inflammation triggered by acidic foods.” “Interstitial cystitis is similar to recurring UTI, with WBC and no bacteria”
Support of online community	Online engagement Seeking advice Symptom sharing Information exchange Self-management strategies Unique populations	“Are these things true or is it a myth?” “The information here to understand my dreadful cycles of UTIs is better than any library.” “Just wondering if anyone has ever gotten or heard of someone that has gotten misdiagnosed with recurrent urine infections when they have other bladder problems.” “Girl, get to the doctor ASAP. UTIs are no joke. They basically travel up the urinary tract and you can get a bladder infection or even a kidney infection. You don't want that. Go to urgent care if you have to. They just need a pee sample. Amoxicillin is your friend!”
Health care utilization	Access barriers Treatment location Patient-physician interactions	“I’m losing a lonely battle with the medical community as these UTIs won’t go away. I’m glad you are here.” “I need to take an action list...no one gives me straight answers.” “If you can't go to the ER due to no insurance, what about a Planned Parenthood? When I was between insurance plans, I went there for UTI's, and all other lady things.” “Mismanagement always happens so recommend seeing a urogynecologist, not a GP.”
Risk factors and prevention	Dehydration Hygiene Anatomical differences Hormonal imbalances Gynecologic factors Comorbid conditions	“Women should be drinking 3L of water per day to prevent UTIs.” “I’ve heard that douching, tampons soaked in different things (tried in the past) can help treat and prevent urine infections.” “Prolapse after pregnancy and ‘abnormal anatomy’ making more prone to infections.”
Antibiotic treatment	Treatment duration Safety and side effects Multidrug-resistant bacteria Effect on microbiome Culture-directed antibiotic treatment	“Prescribing antibiotics for UTIs seems weird.” “It [antibiotic] slayed me! In bed, exhaustion and major aches like I had the flu.” “I’ve done a 5-day course of antibiotics…no improvement” “You get better improvement with antibiotics for 7 days with no sex.”
Alternative therapies	Homeopathy Dietary modifications	“Homeopathic medicine…is more tailored to you, they spend an hour talking to you and can treat your urine infection.” “D-mannose can be used as a treatment or preventatively and I think would be most useful for your mom since it works by sort of lubricating the urinary tract, so the bacteria are unable to stick on and cause inflammation.” “Infections damage bladder lining. recommend drinking baking soda.”

^a^UTI: urinary tract infection.

^b^IC: interstitial cystitis.

#### Quality of Life

The first theme was the quality-of-life burden associated with UTI episodes. The impact on women’s sexual health was frequently mentioned in the context of limiting intercourse due to aggravating symptoms (pain) and managing postcoital antibiotic use. Women described significant negative emotions and hopelessness when they sought self-management strategies and medical care. Self-blame was central to the negative emotions described, as women searched for inherent personal factors causing repetitive infections. Fear of worsening symptoms and progression to pyelonephritis was frequently mentioned.

#### Knowledge Acquisition

Patient knowledge acquisition was another major theme. Based on the keyword content of the posts, it appeared that women consulted online resources at different time intervals to supplement their decision-making while experiencing UTI symptoms or seeking medical care. Some users focused on identifying a differential diagnosis and a specific etiology, while others described self-blame. There was a lack of consensus regarding the optimal work-up and management of UTIs, as evidenced by people providing inconsistent advice to each other on these forums. The misdiagnosis of rUTIs and interstitial cystitis due to symptom overlap, delayed referral, and perceived lack of physician knowledge appeared frequently.

#### Online Community Support

The value and gratitude expressed for the support provided by online communities was another identified theme. In addition to the plethora of information exchanged, including symptom sharing and lay recommendations, we identified geriatric patients, pregnant women, and those with rUTIs as unique populations who frequently appeared as the subject matter of posts with special considerations. Pregnant women had specific interests regarding antibiotic safety and the development of pyelonephritis.

#### Health Care Utilization

The third theme was health care utilization with subthemes centering on the contextual factors influencing whether or not people sought care. Posts included concerns about minimal insurance coverage or being uninsured. Additionally, multiple medical visits for recurrent infections appeared to cause fatigue, frustration, and loss of work productivity. Furthermore, the perceived lack of illness clarity and lack of cure affected the way users commented about their experience.

#### Risk Factors and Prevention

Risk factors and prevention was another theme we identified. Women sought to understand their respective predisposing contributions due to day-to-day activities. The appropriate preventive hydration level was frequently mentioned, with various levels ranging from 1 to 3 L of water. Additionally, genital hygiene (self and partners’) practices were discussed. Pelvic organ prolapse and vaginal atrophy were perceived to increase the risk of UTIs. Diabetes and dementia were also frequently mentioned risk factors. Gynecologic factors that were discussed included methods of contraception and menstrual cycle sanitation products.

#### Antibiotic Treatment and Alternative Therapies

Treatment of UTIs with antibiotics was another identified theme. The appropriate duration and variation in the prescribed length of treatment were discussed, as were the safety and side effects of antibiotics for pregnant and nonpregnant women. The online community misunderstood antibiotic resistance as a patient characteristic that developed, rather than as a bacterial phenomenon. Recommendations to restore the natural gut microbiome were exchanged. The final identified theme was alternative therapies beyond antibiotics to self-manage symptoms, ameliorate current infections, or prevent further UTIs. Some of these alternative therapies included bacteriophage therapy, cranberry products, d-mannose, vitamin C, probiotics, bladder instillation of hyaluronic acid, and oral activated charcoal treatments.

## Discussion

### Principal Results

Our ethnographic study of social media posts on UTIs revealed information on illness experience, lay knowledge, and concerns among women. Unlike prior qualitative studies, we presented patient perspectives that are likely more diverse and candid than data gathered from specialty clinics [5-7]. We found a strong online community support network created via forums to exchange information among peers, which may have partially resulted from frustrations and challenges with medical care. We found that UTIs cause a significant burden on daily activities and, as a result, women engage in supportive conversations about physician interactions, antibiotics, alternative therapies, risk factors, and prevention strategies to bridge knowledge gaps and obtain reassurance from peers.

We captured broad and diverse patient experiences using two methods to conduct digital ethnography. However, our inductive hand coding provided more granular details as expected from directly analyzing quotes, which helped us comprehend online discussions and women’s perspectives for specific themes. For example, culture-directed antibiotic treatment was a unique patient concern identified with our hand coding of posts that was not found using LDA. Although the LDA word clouds consistently represented quality-of-life concerns, hand coding provided examples of fears women faced. Half of the LDA themes related to community support and identifying risk factors and prevention strategies, which was consistent with our hand-coding results.

### Comparison With Prior Work

Ghouri et al [13] previously conducted 15 telephone interviews with women with prior documented UTI and described feelings of hopelessness and lack of support in this group. Our study highlights the support experienced by patients with UTI who exchanged information online. Our findings support prior results that social media is an integral part of processing medical information and can facilitate patient engagement for the exchange of condition-specific knowledge [14,15]. Additionally, it has been widely documented that online forum discussions allow for better intake and information processing [16-18].

Our study was broad, capturing different populations of women. This better allowed the analysis to be guided by the direct, anonymous discussions of patients, making it more likely to be generalizable to the UTI population at large. Prior work only surveyed patients recruited from clinical settings, those with rUTI, and pregnant women [5-7]. Our work, on the other hand, captured the perspectives of several populations in a single analysis, including pregnant women, geriatric women, and women with rUTI across a collection of websites. Geriatric patients, pregnant women, older women, and women with rUTIs were frequently identified in posts with unique concerns, suggesting the need for more targeted outreach to these special populations.

Unlike prior online studies, our study design has the advantage of analyzing multiple websites [5,6]. Flower et al [5] conducted an analysis of 1 online self-help forum for patients with rUTI to understand how women manage their rUTIs. Our findings of alternative therapies, antibiotic concerns, and patient-physician interactions were similar. However, due to the broader sample size, we found other alternative therapies not previously described, additional antibiotic concerns (eg, treatment duration, human microbiome, bacterial resistance, and culture-guided treatment), and more complex health care barriers (eg, specialty care access, insurance coverage, and presumed level of care required for treatment). To our knowledge, the theme of risk factors and prevention has not been previously described in prior UTI online forum literature. Additionally, our findings that pregnant women were concerned about the progression of cystitis to pyelonephritis, as well as the effect of antibiotic use (or lack of treatment) on fetal development, were consistent with those previously found in a study analyzing online content to understand UTIs and antibiotic use in the pregnant population [6].

The semistructured interview style of many qualitative studies may limit and potentially narrow the scope of what patients share in clinical settings. One qualitative one-on-one interview study of 21 women recruited from a larger primary care trial found that patients wanted clinicians to address quality-of-life impact and that they were receptive to the strategy of antibiotic delay, which allows for 48 hours to reassess if infection symptoms subside before starting antibiotics [7]. Women who enrolled in this randomized study were, by default, receptive to different management strategies. Although we found similar quality-of-life concerns, we also identified self-blame, mismanagement in the primary care setting, delayed referrals, inconsistent counseling about treatment guidelines, unmet expectations, and the practice of culture-directed antibiotic use, which were not previously characterized.

Online discussion points were in agreement with the 2019 American Urological Association’s guidelines for uncomplicated rUTI, which recommend first-line antibiotic agents and promote culture-directed antibiotic treatments rather than empiric treatment [19]. This suggests that many patients with UTIs were well educated on the topic. However, there were inconsistencies discussed online for the role of cystoscopy and upper tract imaging, despite the recommendations provided in the updated guidelines to avoid those diagnostic studies in uncomplicated cases [19]. This may have been due to the fact that our data were collected before the publication of the updated guidelines. Concerns about antibiotic collateral damage mentioned in the literature and discussed in the rUTI guidelines were also supported by our analysis [19].

### Limitations

Despite the innovation and patient inclusivity of our study, there are important limitations that can inform future work. We did not have access to demographic information, and the website content could have been restricted by the sample of websites accessed by Treato and our search strategy. Although we focused our analysis on women, it is possible that some men participated on the forums and were included in the analyses. The anonymous data may also contribute to patient misclassification since we cannot confirm a diagnosis, but our best attempt was made using contextual factors. Additionally, our analysis and conclusion relied on the degree to which individuals post online. We could not characterize specific posts’ engagement level, such as individual read and reply counts. Our study, by default, excluded those women who do not exchange medical information on the internet.

### Conclusions

Digital ethnography combining qualitative analysis and LDA allowed us to analyze free-range patient perspectives, which are currently not found in the UTI literature. First, unlike focus group studies, anonymity is a clear driver of candid, honest conversations, facilitating online users to provide support and address the most important concerns. Second, there was a pervasive element of fear: fear of not treating UTIs, as well as fear of the sequelae associated with antibiotic treatments. Finally, the use of online forums empowered women to self-manage their condition and take their care into their own hands. Our findings also demonstrate the reliability of using online social media data to learn about patient behavior and decision-making, which is important to guide how we engage with patients and disseminate society-sponsored guidelines. Patient information, outreach, and treatment guidelines by medical societies must be congruent with patients’ concerns. Physicians can use this data to discuss misconceptions and improve patient-centered care.

## Appendix

Multimedia Appendix 1 Search and exclusion terms used in the Treato algorithm.

## Abbreviations

LDA: latent Dirichlet allocation
PLUS: Prevention of Lower Urinary Tract Symptoms
rUTI: recurrent urinary tract infection
UTI: urinary tract infection
