# Supplementary figures and images for: Using Large-scale Social Media Analytics to Understand Patient Perspectives About Urinary Tract Infections: Thematic Analysis
Source: J Med Internet Res. 2022 Jan 25;24(1):e26781. doi: 10.2196/26781 (PMC8826307; doi:10.2196/26781)

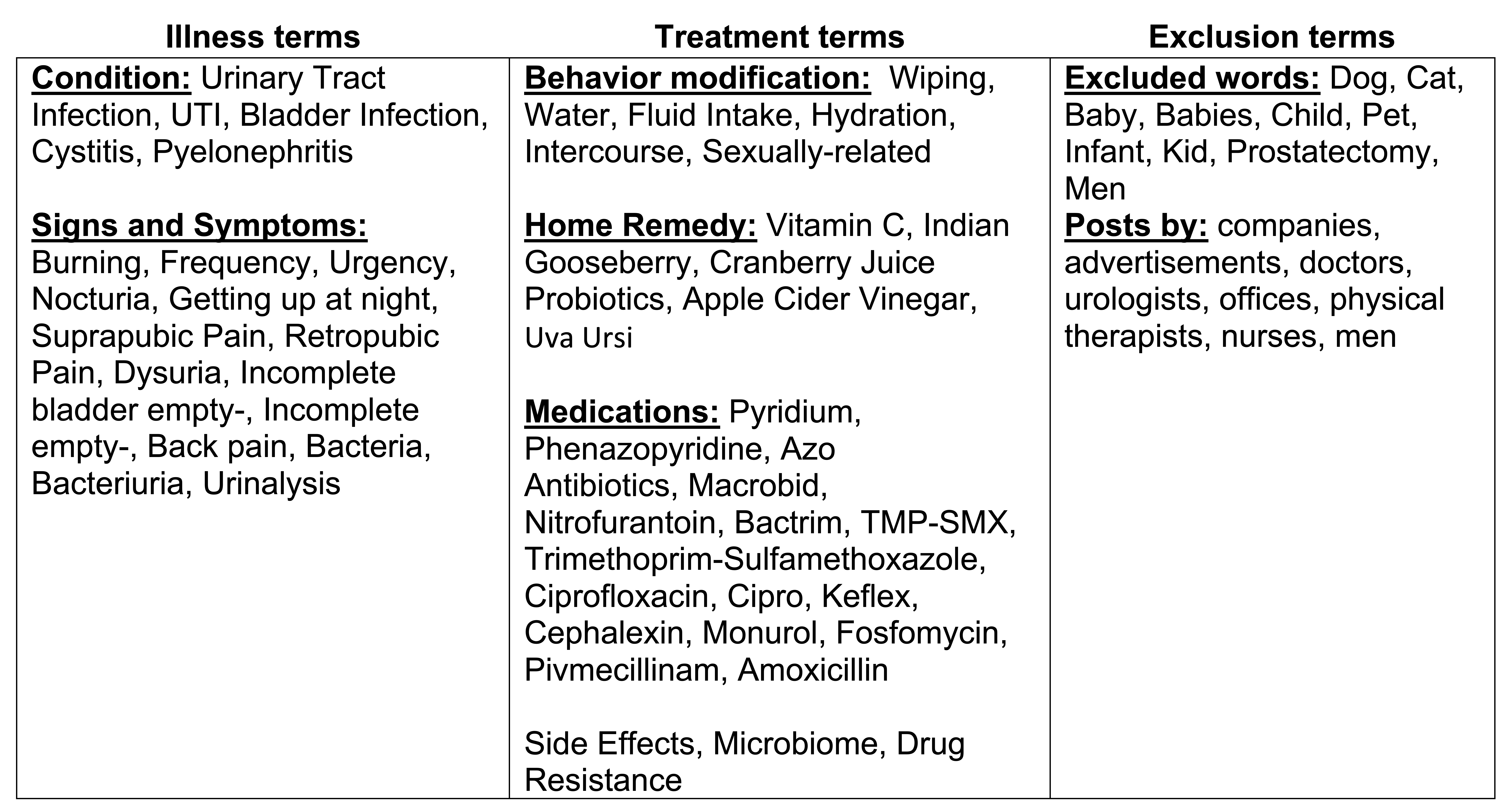

Supplement: Multimedia Appendix 1 [file jmir_v24i1e26781_app1.png]
